# Polarization-Sensitive and Wide Incidence Angle-Insensitive Fabry–Perot Optical Cavity Bounded by Two Metal Grating Layers

**DOI:** 10.3390/s20185382

**Published:** 2020-09-20

**Authors:** Jehwan Hwang, Zahyun Ku, Jiyeon Jeon, Yeongho Kim, Deok-Kee Kim, Eun Kyu Kim, Sang Jun Lee

**Affiliations:** 1 Interdisciplinary Materials Measurement Institute, Korea Research Institute of Standards and Science, Daejeon 34113, Korea; wowhwang87@gmail.com (J.H.); jeony210@gmail.com (J.J.); ykim172@kriss.re.kr (Y.K.); 2 Department of Physics and Research Institute for Convergence of Basic Sciences, Hanyang University, Seoul 04763, Korea; 3 Materials and Manufacturing Directorate, Air Force Research Laboratory, WPAFB, OH 45433, USA; zahyun.ku.1.ctr@us.af.mil; 4 Department of Electronic Engineering, Sejong University, Seoul 05006, Korea; deokkeekim@sejong.ac.kr

**Keywords:** polarimetric imaging, subwavelength grating, Fabry–Perot cavity, surface plasmon resonance, double-layer metal grating

## Abstract

Infrared (IR) polarimetric imaging has attracted attention as a promising technology in many fields. Generally, superpixels consisting of linear polarizer elements at different angles plus IR imaging array are used to obtain the polarized target signature by using the detected polarization-sensitive intensities. However, the spatial arrangement of superpixels across the imaging array may lead to an incorrect polarimetric signature of a target, due to the range of angles from which the incident radiation can be collected by the detector. In this article, we demonstrate the effect of the incident angle on the polarization performance of an alternative structure where a dielectric layer is inserted between the nanoimprinted subwavelength grating layers. The well-designed spacer creates the Fabry–Perot cavity resonance, and thereby, the intensity of transverse-magnetic I-polarized light transmitted through two metal grating layers is increased as compared with a single-layer metal grating, whereas transverse-electric (TE)-transmitted light intensity is decreased. TM-transmittance and polarization extinction ratio (PER) of normally incident light of wavelength 4.5 μm are obtained with 0.49 and 132, respectively, as the performance of the stacked subwavelength gratings. The relative change of the PERs for nanoimprint-lithographically fabricated double-layer grating samples that are less than 6% at an angle of incidence up to 25°, as compared to the normal incidence. Our work can pave the way for practical and efficient polarization-sensitive elements, which are useful for many IR polarimetric imaging applications.

## 1. Introduction

Infrared (IR) polarimetric imaging systems have been extensively employed in many fields, such as remote sensing, military applications, and environmental protection [1,2,3,4,5]. Polarimetric images provide information based on the polarization signature of radiation instead of detecting optical radiation with scalar fields in traditional infrared images [6,7]. The polarization signature depends on the polarization properties of scattering, reflection, and absorption, which result from the geometry and roughness of the target surfaces, and it can be used to distinguish the target from the clutter and background [8,9,10,11,12]. The polarimetric imaging system usually uses a broadband imager for the spatiotemporal information with the mechanical parts of optical components, to obtain the polarimetric signature of a target. However, these mechanical parts make the acquisition of real-time image information difficult, as well as increase the cost and complexity of imagers.

Division-of-focal-plane (DoFP) polarization imaging sensors have been widely studied because of their capability to acquire both the intensity and polarization information in real-time using a monolithically integrated focal plane array (FPA) with a micro-optical element (array of micropolarizers) [13,14,15,16]. Typically, the micropolarizer array consists of a 2 × 2 array of metallic subwavelength gratings oriented at 0, 45, 90, and 135° to simultaneously determine the first three Stokes parameters [17,18,19,20]. Metallic subwavelength gratings are one-dimensional periodic structures with much smaller than the wavelength of the light, which have been recognized as an effective polarizer candidate for the IR regime [21,22,23,24]. Specifically, a large portion of linearly polarized incident light perpendicular to the grating direction (TM-, *p*- or transverse-magnetic polarization) can be transmitted through metallic gratings, while the incident light with polarization parallel to the grating direction (TE-, *s*- or transverse-electric polarization) is rarely transmitted. The polarization extinction ratio (PER) can be used as the performance indicator of designed polarizers and is defined as the ratio of the transmitted light intensity of perpendicular polarizations (i.e., TM- and TE- polarization).

Recently, a double-layer grating structure has been proposed to improve the polarization detection performance, as well as to reduce the complexity and cost of a lithography technology resulting from simply decreasing the grating period in a single-layer grating structure. As compared to a single-layer grating, the double-layer grating was demonstrated to enhance TM-transmission owing to the multiple reflections, Fabry–Perot (FP) cavity resonance, inside a dielectric spacer layer interposed between two metal gratings, on the other hand, TE-transmitted light intensity through the double-layer grating tends to drastically decrease, leading to a higher PER [25,26,27,28].

As mentioned earlier, one of the main advantages of DoFP polarization sensors is the capability of capturing polarization information at every frame by incorporating a pixel-wise polarizer array aligned to an FPA; however, this hybridized configuration can also bring the disadvantages [29,30,31], such as a loss of spatial resolution (the spatial resolution is decreased by a factor of the number of linear polarizer elements that constitute the superpixel) and inaccuracy of the captured polarization information (each pixel has a varying instantaneous field of view (IFoV), and thereby, it is worth investigating the margin of the validity of a double-layer grating structure used as the linear polarizer element in terms of the angle of incidence within the FoV range). Note that various interpolation methods have been recently proposed to improve both the spatial resolution and the accuracy of the polarization information, due to the sensors’ spatially modulated arrangement of a micro-polarization array. These include bilinear, bicubic, bicubic spline, and gradient-based interpolation methods [32,33,34], new micro-polarizer array patterns-used interpolation method [35,36], spatio-temporal channeled approach [37], smoothness-based interpolation method [38], Newton’s polynomials based interpolation [39], correlation-based interpolation method [40], deep convolutional neural network-based polarization demosaicing [41,42], minimized Laplacian polarization residual interpolation [43], sparse representation-based demosaicing method [44], etc.

Here, we focus on experimentally and theoretically analyzing the polarization sensitivity and PER of the double-layered Au grating using the polar and azimuthal angles of incident light on the basis of FoV of the thermal imaging lens. Numerical simulations based on the finite integration technique and the finite element method are carried out to better understand the resonance behavior of the double-layer Au grating, specifically, surface plasmon resonance at the interface between gold and substrate (when stacking the grating layers with a large grating period) and Fabry–Perot resonance. The analysis and modeling results show a good agreement with Fourier transform infrared (FTIR)-measured data of the nanoimprint-lithographically fabricated double-layer Au grating.

## 2. Design of Double-Layer Grating Using a Multiple-Layer Model

**Figure 1:**
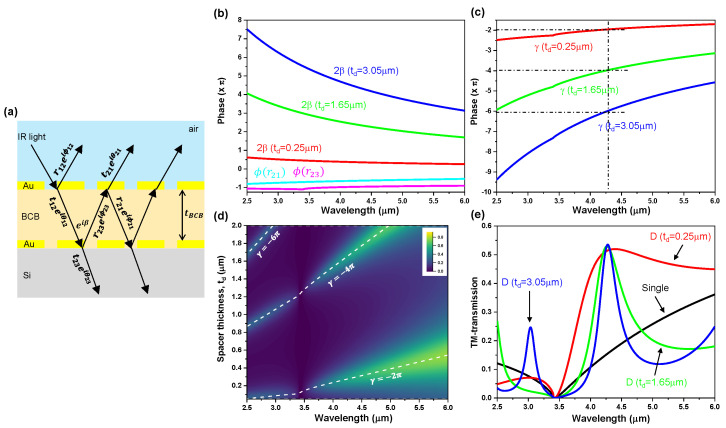
(**a**) Schematic illustration of the multiple-layer model of the double-layer grating (grating period *p* = 1.0 μm; grating width *w* = 0.7 μm; *t*_Au_ = 0.1 μm); (**b**) phase terms, $\phi \left(r_{21}\right)$, $\phi \left(r_{23}\right)$, 2β, used in the Fabry–Perot (FP) cavity resonance condition γ; (**c**) $\gamma =\phi \left(r_{21}\right)+\phi \left(r_{23}\right)-2\beta$; (**d**) transverse-magnetic I-transmission spectra colormap $T\left(\lambda ,t_{d}\right)$ obtained by the analytical calculation using the multiple-layer model; (**e**) reconstructed TM-transmission (based on the multiple-layer model) for the double-layer grating with a spacer thickness of 0.25 μm, 1.65 μm, 3.05 μm. The FP cavity resonance condition of γ=−2π, −4π, −6π is satisfied at λ = ~4.3 μm.

**Figure A1:**
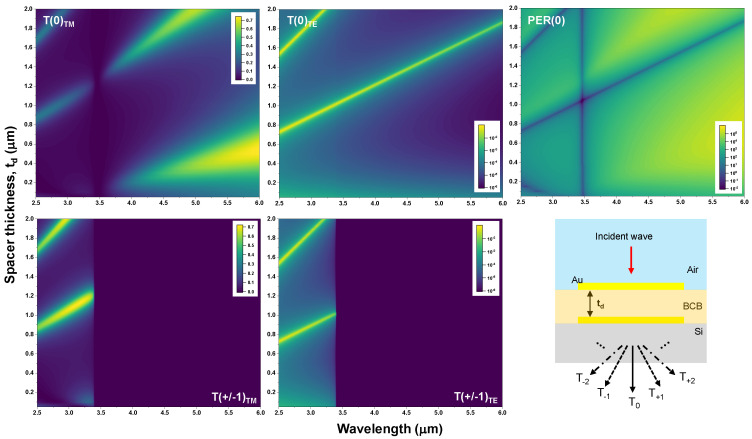
Colormaps of simulated zeroth and first diffracted transverse-magnetic (TM)-, transverse-electric (TE)-transmission, polarization extinction ratio as a function of wavelength *λ* and spacer thickness *t*_d_ ranging from 0.05 μm to 2 μm with a step of 10 nm. Note that the grating period, grating fill factor, Au thickness, and spacer thickness were fixed at *p* = 1.0 μm, *r* = 0.7, and *t*_Au_ = 0.1 μm, respectively.

A multiple-layer model [45,46,47] was employed to efficiently design the double-layer grating structure and to better understand the underlying mechanism of wave propagation inside the spacer layer bounded by two identical gold (Au) grating layers as illustrated in Figure 1a. The overall reflection and transmission coefficients of the double-layer grating can be obtained by a transfer matrix model, which depends on the transmission and reflection of each grating layer. Using the multiplication of the transfer matrix of each layer (i.e., upper and lower grating layers, and spacer layer), the overall transmission coefficient *t* can be obtained as given by $t=t_{12}t_{23}e^{-i\beta }/\left(1-r_{21}r_{23}e^{-2i\beta }\right)$ where $r_{21}$ and $r_{23}$ are the reflection coefficients at the backside of upper Au grating and front side of lower Au grating, respectively, and $t_{12}$ and $t_{23}$ are the transmission coefficients through the upper Au grating and lower Au grating, respectively. $\beta =n_{spacer}\cdot k\cdot t_{d}$, where $n_{spacer}$ is the refractive index of spacer, k is the wave vector in free space, $t_{d}$ is the spacer thickness (Figure 1b). The refractive index of the spacer material used for the multiple-layer model was taken as $n_{spacer}=$ 1.54, which is close to the measured real part of the refractive index of benzocyclobutane (BCB). Here, to create the Fabry–Perot (FP) cavity resonance inside the spacer of the double-layer grating, the round-trip propagation phase condition $\gamma =\phi_{r_{21}}+\phi_{r_{23}}-2\beta$ where $\phi_{r_{21}}$ and $\phi_{r_{23}}$ are the phase of $r_{21}$ and $r_{23}$, respectively) is needed to be equal to an integer multiple of 2π (Figure 1c). Figure 1d shows the TM-transmission colormap, $T\left(\lambda ,t_{d}\right)$, obtained by the analytical calculation using the multiple-layer model, and the white dash lines indicate where the FP cavity resonance condition for TM-polarized incidence is satisfied (i.e., γ=−2π, −4π, −6π). In the wavelength of interest (mid-wavelength infrared region, MWIR, 3−5 μm), the spectral position of TM-transmission peaks for the double-layer grating ($T{\left(0\right)}_{TM}$ in Figure A1) agrees well with the position of FP cavity resonances (Figure 1d). γ=−2π (the lowest order of FP cavity resonance) and $t_{d}=$ 0.25 μm are the most suitable to realize the broadband linear polarizer covering the MWIR range. Narrowband transmission can be found at the high order FP cavity resonance, as shown in Figure 1d,e, and Figure A1 (an increase in the spacer thickness produces more transmission peaks and makes the transmission bandwidth narrower).

## 3. Theoretical Analysis of Incidence Angle-Dependent Polarization Sensitivity and PER of Double (Single)-Layer Au Grating 

**Figure 2:**
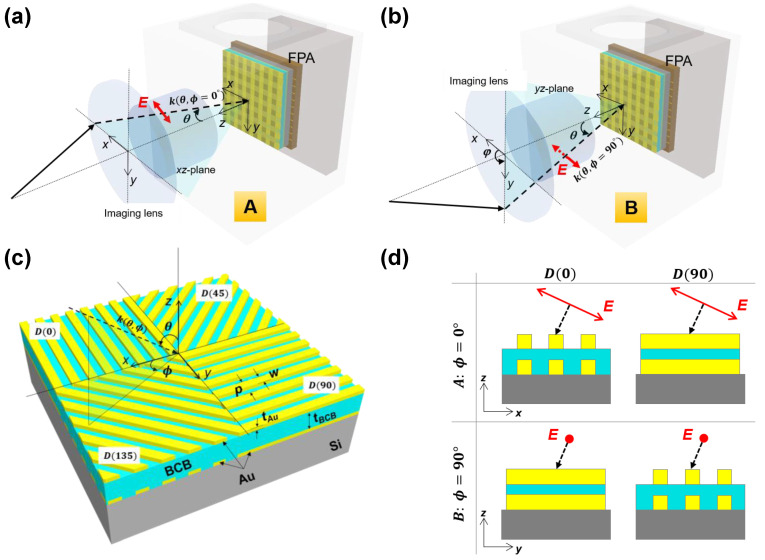
Schematic of the polar (θ) and azimuth (ϕ ) angle-dependent infrared (IR) incidence through the thermal imaging lens: Illustrations of (**a**) test scenario A; (**b**) test scenario B; (**c**) three-dimensional configuration of the superpixel consisting of linear polarizer elements at different angles (0, 45, 90, 135°). k, θ, and ϕ denote the incident wave vector, polar angle, and azimuthal angle, respectively; (**d**) *D* (0) and *D* (90) for (θ, ϕ = 0 or 90°).

**Figure A2:**
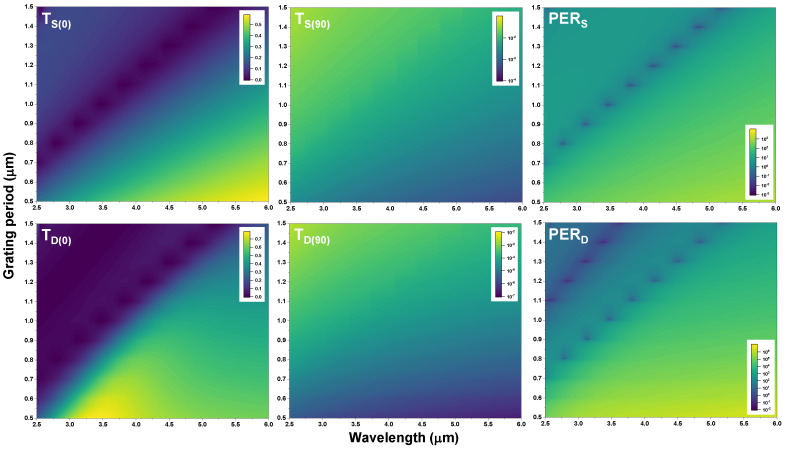
Colormaps of simulated transmission ($T_{x\left(y\right)}$, where *x* = S (single-layer Au grating) or D (double-layer grating), and *y* = incidence angle) and polarization extinction ratio ($PER_{x}=T_{x\left(0\right)}/T_{x\left(90\right)}$, where *x* = S or D) of the single- and double-layer Au gratings as a function of wavelength (*λ*) and grating period (*p*). Note that the ratio of grating width to grating period (*r* = *w*/*p*), gold thickness (*t*_Au_), and spacer thickness (*t*_d_) were fixed at *r* = 0.7, *t*_Au_ = 0.1 μm, and *t*_d_ = 0.25 μm, respectively.

**Figure A3:**
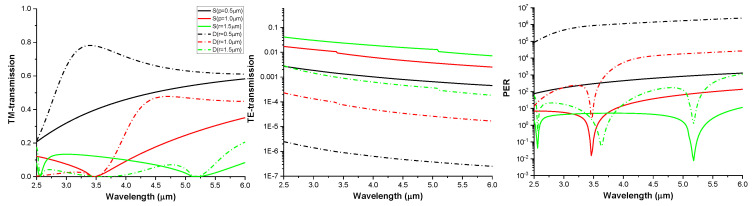
Simulated transmission and polarization extinction ratio of the single- and double-layer Au gratings for the grating period *p* = 0.5, 1.0, 1.5 μm.

**Figure A4:**
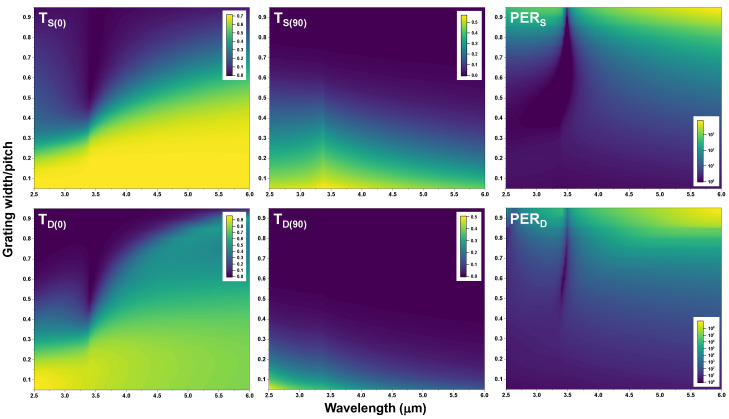
Colormaps of simulated transmission ($T_{x\left(y\right)}$, where *x* = S (single-layer Au grating) or D (double-layer grating), and *y* = incidence angle) and polarization extinction ratio ($PER_{x}=T_{x\left(0\right)}/T_{x\left(90\right)}$, where *x* = S or D) of the single- and double-layer Au gratings as a function of wavelength and ratio of grating width to grating period (*r* = *w*/*p*). Note that the grating period, Au thickness, and spacer thickness were fixed at *p* = 1.0 μm, *t*_Au_ = 0.1 μm, and *t*_d_ = 0.25 μm, respectively.

**Figure A5:**
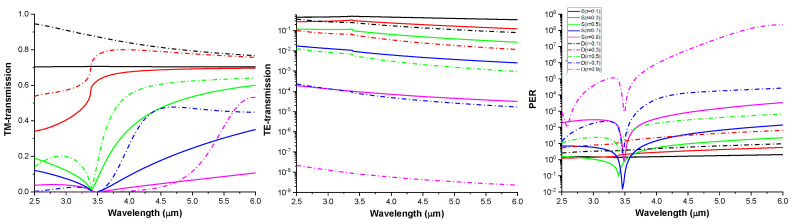
Simulated transmission and polarization extinction ratio of the single- and double-layer Au gratings for *w* = 0.1, 0.3, 0.5, 0.7, 0.9 μm (*r* in a unit of um can be considered as the width because *p* is fixed at 1.0 μm).

To create test scenarios (for the study of incidence angle-dependent polarization sensitivity and PER of single- and double-layer Au grating structures), we first assumed that the superpixel is composed of a 2 × 2 array of single- or double-layer Au gratings oriented at 0 (perpendicular to $\hat{x}$), 45, 90 (parallel to $\hat{x}$), and 135° as illustrated in Figure 2. Note that the geometrical parameters (grating period *p*, grating width *w*, Au thickness *t*_Au_) of the single- and double-layer Au gratings are fixed at 1 μm, 0.7 μm, 0.1 μm, respectively, and a dielectric spacer (*t*_BCB_ = 0.25 μm) was used to separate two Au grating layers in the double-layer structure (Figure 1, Figure A1, Figure A2, Figure A3, Figure A4 and Figure A5). Moreover, we specified two test scenarios to mimic the angles of linearly polarized IR incidence passing through the thermal imaging lens as follows: k(θ,ϕ=0°) and k(θ,ϕ=90°), incident IR rays in a direction described by the spherical coordinates, are characterized by the electric field lying in the plane of incidence (*xz*-plane) for test scenario A as illustrated in Figure 2a,d, and the electric field perpendicular to the plane of incidence (*yz*-plane) for test scenario B as indicated in Figure 2b,d, respectively.

**Figure 3:**
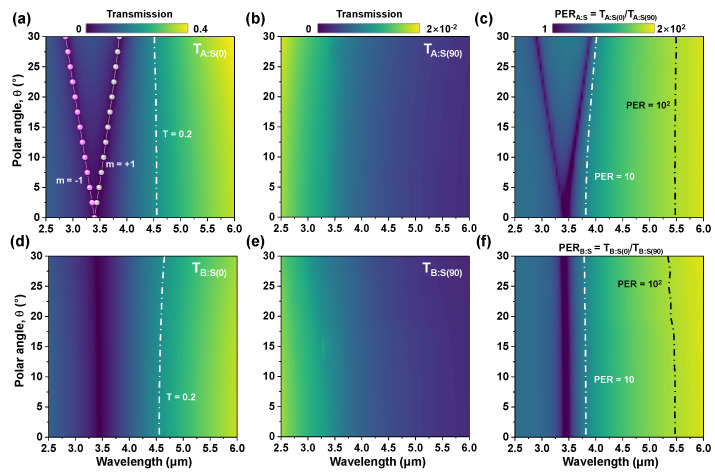
Simulated transmission and polarization extinction ratio of the single-layer Au grating as a function of wavelength *λ* and the polar angle *θ* for (**a**–**c**) test scenario A (i.e., $T_{A:S\left(0\right)},\;T_{A:S\left(90\right)},\;PER_{A:S}$); (**d**–**f**) test scenario B (i.e., $T_{B:S\left(0\right)},\;T_{B:S\left(90\right)},\;PER_{B:S}$ ). The solid lines with symbols represent the wavelengths of the excited surface plasmon resonance (SPR) modes, $\lambda_{sp}\left(\theta ,\phi \right)$, calculated by Equation (2). Note that the SPR mode with *m* = −1 (red) and +1 (black).

We performed 3D full field EM simulation (CST Microwave Studio [48]) to theoretically analyze the polarization sensitivity and PER of double-layer (*D*) and single-layer (*S*) gold grating structures using the angled incidence. The optical parameters for constitutive materials (i.e., BCB and silicon (Si) are used as a spacer layer and the substrate, respectively) were taken as $n_{BCB}$ = 1.54, $n_{Si}$ = 3.4, and a simple Drude model was used for gold permittivity (with plasma frequency of 9.02 eV and scattering frequency of 0.038 eV) [49]. Figure 3 shows the simulated transmission ($T_{x:y}$, where *x* = test scenario A or B; *y* = an element in the superpixel) and the polarization extinction ratio ($PER_{x:S}=T_{x:S\left(0\right)}/T_{x:S\left(90\right)}$) for the single-layer Au grating. The transmission dips at the incidence angle θ of 0° for test scenario A (ϕ=0°) and B (ϕ=90°) are observed at ~3.4 μm, as shown in Figure 3a,d, which correspond to the first-order surface plasmon resonance (SPR) at the interface between Au grating and Si substrate. As the polar angle of the incident light is increased, this dip at 3.4 μm is split into two dips at ~2.9 μm and ~3.9 μm in case of θ=30°,ϕ=0°, as indicated in Figure 3a, whereas the simulation result for test scenario B (θ,ϕ=90°) shows no splitting, as shown in Figure 3d. The momentum matching condition was used to understand the underlying mechanism of the SPR-splitting when a linearly polarized light is incident obliquely.

(1) $${\vec{k}}_{sp}={\vec{k}}_{∥}\pm m{\vec{G}}_{x}$$

(2) $$\lambda_{sp}=\frac{p}{m}\left(-sin\theta cos\phi \pm \sqrt{sin^{2}\theta \left(cos^{2}\phi -1\right)+n^{2}}\right)$$

where ${\vec{k}}_{sp}$ and $\lambda_{sp}$ are the SPR wave vector and wavelength, ${\vec{k}}_{∥}$ is the in-plane wave vector of the incident radiation, ${\vec{G}}_{x}$ is the reciprocal vector of the one-dimensional grating ($\left|{\vec{G}}_{x}\right|=2\pi /p$, where *p* is the grating period), *m* is an integer representing the coupling order of SPR, θ and ϕ are the polar and azimuthal angles, and *n* is the refractive index of the silicon substrate.

In Figure 3a, the 2-fold degenerate (±1) mode for normal incidence (θ=0°) is split into two individual SPR modes, (+1) and (−1) at $2\pi /{\vec{k}}_{\left(+1\right)}$ and $2\pi /{\vec{k}}_{\left(-1\right)}$, respectively, because ${\vec{k}}_{\left(+1\right)}$ and ${\vec{k}}_{\left(-1\right)}$ have unequal magnitudes when θ≠0°. However, the 2-fold degenerate (±1) mode for test scenario B ($T_{B:S\left(0\right)}$) is not split, due to only a very minor increase in the magnitude of ${\vec{k}}_{\left(\pm 1\right)}$ against the polar angle. Note that the electric field perpendicular to the Au gratings lies in the *xy* plane. Instead, the degenerate (±1) mode is slightly blue-shifted by $\sqrt{n^{2}-sin^{2}\theta }$ as the incident light comes in the polar angle (θ>0°). Figure 3b,e shows the simulated transmission of the single-layer Au gratings oriented at 90° with polar angles for test scenario A and B ($T_{A:S\left(90\right)}$ and $T_{B:S\left(90\right)}$). $T_{A:S\left(90\right)}$ and $T_{B:S\left(90\right)}$ are found to be nearly independent of the incidence angle (θ). Figure 3c,f clearly shows the trend of the calculated PERs, which are closely similar to $T_{A:S\left(0\right)}$ and $T_{B:S\left(0\right)}$.

**Figure 4:**
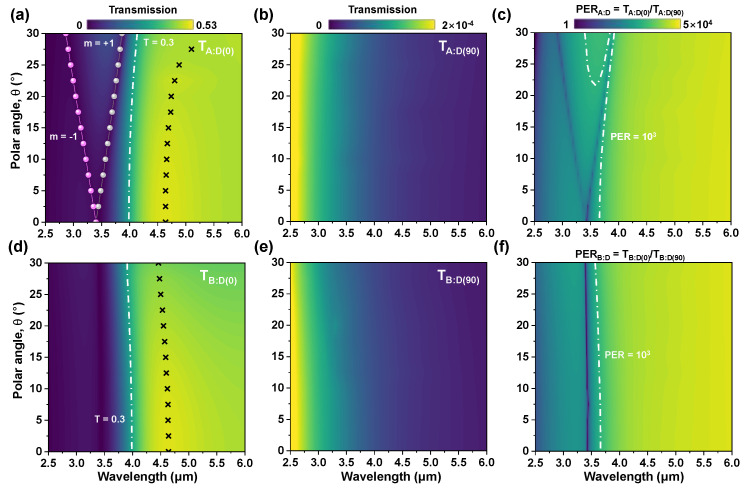
Colormaps of the simulated transmission and polarization extinction ratio of the double-layer Au grating structure for (**a**–**c**) test scenario A; (**d**–**f**) test scenario B, which are presented in color as a function of wavelength λ and the incidence angle θ.

Figure 4a shows the simulated transmission of an alternative grating structure where a dielectric spacer layer is inserted between the upper and lower Au grating layers, i.e., the double-layer Au grating structure ($T_{A:D\left(0\right)}$). We observed the transmission dip at the same wavelength of single-layer grating (~3.4 μm, Figure 3a), which is attributed to the SPR excited at the interface of lower Au grating and Si substrate in the double-layer structure. As the polar angle increases from 0 to 30°, (+1) SPR mode red-shifts from 3.4 μm to 3.9 μm, and its transmission intensity changes. On the contrary, an FP cavity mode for normal incidence is created at ~4.5 μm, which is due to the multiple reflections inside the spacer bounded by two SPR layers. As the polar angle θ increases from 0 to 20°, the FP resonance peak (cross-symbol) is slightly red-shifted by 2.16% or less, and the transmission intensity at θ=20° is comparable to θ=0° (Note that the wavelength of FP-peak increases rapidly at larger incidence angles). When the incidence angle is varied (θ=0°~20°), the overall transmission intensity and FP resonance peak for test scenario B is slightly reduced and blue-shifted (Δ*λ* < ~90 nm), respectively, as shown in Figure 4d. Figure 4b,e shows that the simulated transmission of the *D* (90) sample is almost unaffected by the polar angle and test scenarios, and is about two orders lower than the single-layer structure. Figure 4c,f shows a high PER of more than ~10^3^ in the region of 4–6 μm, regardless of polar angles.

## 4. Polarization Sensitivity Measurement of NIL-Fabricated Double (Single)-Layer Grating

**Figure 5:**
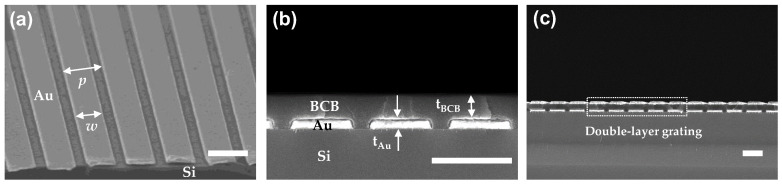
Scanning electron microscope images (tilted- and cross-sectional views) of (**a**) NIL-fabricated lower Au gratings (single-layer); (**b**) benzocyclobutane (BCB)-spin coated on the single-layer Au gratings; (**c**) double-layer Au gratings, followed by NIL-processed upper Au gratings on BCB atop the single-layer grating. The fabricated structural parameters are *p* = 1.0 μm, *w* = 0.7 μm, *t*_Au_ = 0.1 μm, and *t*_BCB_ = 0.25 μm. All scale bars are 1 μm.

Polymethyl methacrylate (PMMA) was spun onto a substrate (Si) as a sacrificial layer for liftoff processing, followed by UV nanoimprint resist. The subwavelength gratings were fabricated by imprinting a polydimethylsiloxane (PDMS) mold. After demolding, an anisotropic plasma-reactive ion etch was used to etch the residual UV resist (CHF_3_/O_2_) and the PMMA (O_2_). Five nanometer-thick Cr and 100 nm-thick Au were deposited by e-beam evaporation, followed by liftoff processing with acetone to remove the PMMA layer (Figure 5a). Next, BCB used as the FP optical cavity was spin-coated on the NIL-fabricated single-layer grating sample, followed by curing at 250 °C for 1 h (Figure 5b). Finally, the upper Au grating was fabricated by using the same NIL process as described above (Figure 5c). FTIR spectroscopy measurements were carried out to confirm the validity of the simulation results, and the polarization extinction ratios (PERs) for test scenario A and B were calculated using the measured transmission spectra (i.e., $PER_{i:j}\equiv T_{i:j\left(0\right)}/T_{i:j\left(90\right)}$, where *i* = test scenario A or B, *j* = single- or double-layer grating). A commercial wire grid polarizer was used to generate the linearly polarized incident light, and the single- and double-layer grating samples were tilted from 0 to 30° to mimic the incidence light through the thermal imaging lens.

**Figure 6:**
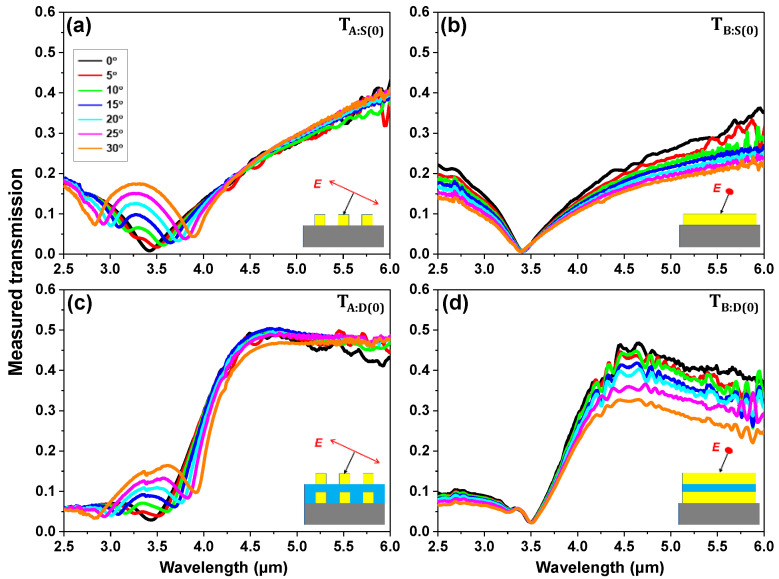
Measured θ-dependent transmission spectra of 0°-orientated (**a**,**b**) single- and (**c**,**d**) double-layer Au gratings for both A, B cases. The fabricated samples were Fourier transform infrared (FTIR)-measured from θ=0° to θ=30° with a step of 5°.

**Figure A7:**
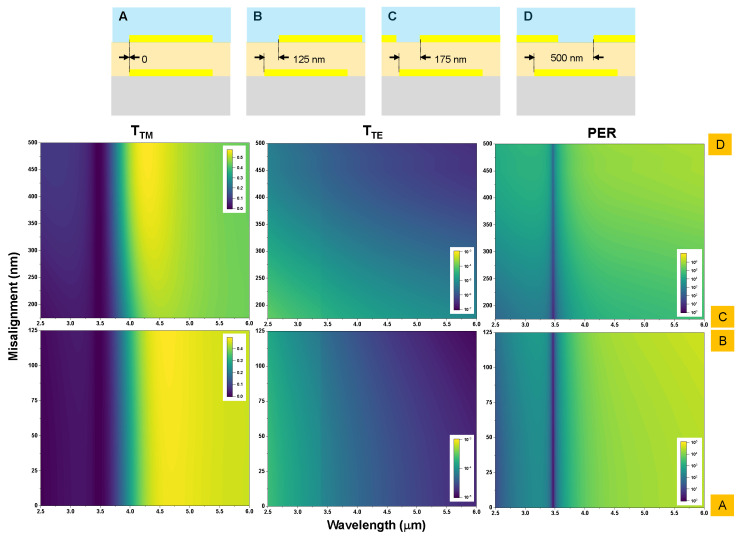
(Upper panel) Schematic view of alignment-shifted top Au grating to bottom Au grating; (Lower panel) colormaps of simulated TM-, TE-transmission, and polarization extinction ratio as a function of wavelength and misalignment between top and bottom Au gratings. The lateral misalignment between two metal gratings is gradually varied from perfectly aligned (i.e., Case A: 0 nm) to completely alignment-shifted top Au grating (i.e., Case D: 500 nm). The geometrical parameters of the double-layer grating are as follows: grating period (*p*) = 1 μm, grating width (*w*) = 0.7 μm, Au thickness (*t*_Au_) = 0.1 μm, spacer thickness (*t*_d_) = 0.25 μm.

**Figure A8:**
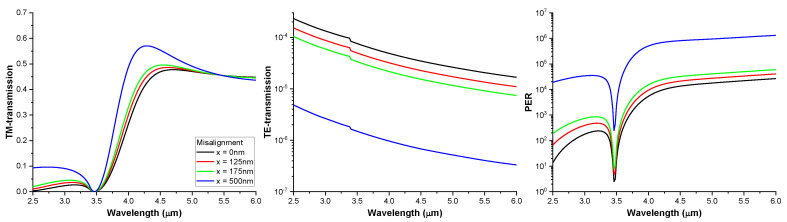
Simulated TM-, TE-transmission, and polarization extinction ratio for alignment-shift of top Au grating (0 nm, 125 nm, 175 nm, 500 nm).

Figure 6 shows the FTIR-measured transmission spectra of the single- and double-layer Au gratings, *S*(0), and *D*(0), using various polar angles for test scenario A and B ($T_{A:S\left(0\right)},\;T_{B:S\left(0\right)},\;T_{A:D\left(0\right)},\;T_{B:D\left(0\right)}$). In test scenario A, the 2-fold degenerate SPR dip of ~3.4 μm (m=±1) is split into 2.84 μm (*m* = −1) and 3.90 μm (m=+1) at the polar angle of 30° for both single- and double-layer Au gratings, as shown in Figure 6a,c. This result agrees well with Figure 3a. In test scenario B, the wavelength of SPR mode is also found to be ~3.4 μm for normal incidence (θ=0°) and it is blue-shifted by Δ*λ* ≈ ~40 nm when the polar angle is increased from 0 to 30° (Figure 6b). Only the double-layer grating sample appears the transmission peak at ~4.5 μm for both scenarios (Figure 6c,d), which originates from FP cavity resonance inside the BCB spacer ($T_{i:D\left(0\right)}$= 0.49 at θ=0°, *I* = A or B), as explained in Section 2. Note that the discrepancy in the peak location of double-layer grating between simulation (Figure 1, Figure 2, Figure 3, Figure 4 and Figure 5) and experiment (Figure 6), Δ ≈ 0.2 μm, is probably due to the imperfections in the fabrication, specifically the misalignment between the upper and lower Au gratings. Detailed information can be found in Appendix A (Figure A7 and Figure A8). 

**Figure 7:**
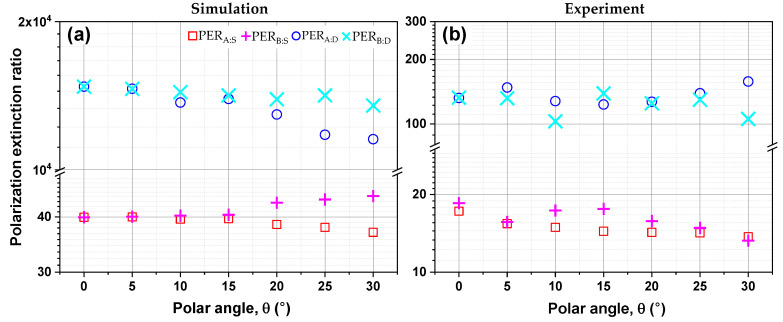
(**a**) Simulated and (**b**) measured-polarization extinction ratios (PERs) of the single and double-layer Au gratings as a function of incidence angle (θ) at *λ*= 4.5 μm.

Figure 7 shows the simulated and measured polarization extinction ratios (PERs) of the single- and double-layer Au gratings, which were calculated at a wavelength of 4.5 μm, corresponding to the FP cavity mode. For test scenario A and B, the simulated PERs of the single- and double-layer Au gratings for the different polar angles were ~40 and 10^4^, respectively, as shown in Figure 7a. The overall agreement between simulated PER and experimental PER at the FP cavity resonance wavelength is apparent from Figure 6 for the incidence angle range we considered. The measured PERs for the single- and double-layer gratings are found to be ~18 and ~132 at normal incidence. By comparison, this corresponds to the polarization sensitivity improvement of the double-layer grating structure by a factor of ~7 times over the single-layer grating structure, which is enabled by the incorporation of a BCB spacer interposed between two metal grating layers. As a result, TM-transmission is enhanced owing to the FP cavity resonance, and simultaneously, TE-transmission is suppressed due to the double-layer grating configuration, i.e., vertically stacked two identical grating layers. Furthermore, the performance indicator for polarization sensitivity, $PER_{i:j}$ (*i* = test scenario A or B, *j* = single- or double-layer grating), in the MWIR regime does not vary with the change of incident angle in some degree. In particular, the relative difference of measured PER for double-layer structure is ~4.7% (θ=15°), ~5.8% (θ=20°), and ~2.2% (θ=25°) as compared to θ=0° (normal incidence).

## 5. Conclusions

We have experimentally and numerically demonstrated the polarization-sensitivity, and incidence angle-insensitivity of a dielectric spacer bounded by nanoimprint-lithographically fabricated two gold subwavelength grating layers. Using the well-designed spacer serves (1) to create the multiple reflections inside, thereby enhancing the transmittance of IR light polarized perpendicular to the grating direction, (2) to separate the two identical grating layers, thereby suppressing the transmittance of IR light polarized parallel to the grating direction, (i.e., reduced light intensity, due to passing through two identical grating layers), (3) to reduce the cost of a lithography technology resulting from simply decreasing the grating period in a single-layer grating structure (when the grating layers with a large grating period are stacked, the polarization sensitivity can be improved by a factor of ~7 times over the single-layer grating structures). More interestingly, the polarization extinction ratio (PER, used as the measure of the polarizer performance) of the double-layer grating structure is insensitive to the incidence angle to some degree ($\left|PER_{\theta }-PER_{\theta =0}\right|/PER_{\theta =0}$ < 6%, θ ≤ 25°). We expect that the results drawn from this work could be used as a basis to improve the overall performance of polarization-sensitive elements (constituting the superpixel) and provide a new perspective for the application of IR polarimetric imaging.

## Figures and Tables

**Figure A6:**
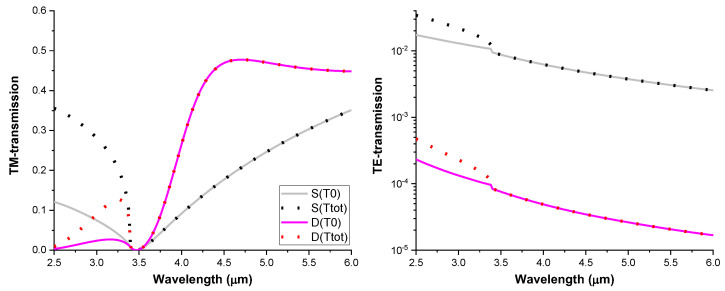
Simulated TM- and TE-transmissions of single-layer grating (S) and double-layer gratings (D) for normal incidence. $T_{0}$ is the zeroth diffracted transmission and $T_{tot}$ is the total transmission, i.e., the sum of transmission in all diffraction orders. The geometrical parameters of the single- and double-layer gratings are fixed at *p* (grating period) = 1 μm, *w* (grating width) = 0.7 μm, *t*_Au_ (Au thickness) = 0.1 μm, and *t*_d_ (spacer thickness) = 0.25 μm.

## Appendix A

Figure A1 shows the zeroth and the first diffracted transverse-magnetic (TM)- and transverse-electric (TE)-transmission ($T{\left(0\right)}_{TM}$,$\;T{\left(0\right)}_{TE}$, $T{\left(\pm 1\right)}_{TM}$,$\;T{\left(\pm 1\right)}_{TE}$), and the polarization extinction ratio (PER) calculated by using $T{\left(0\right)}_{TM}$ and$\;T{\left(0\right)}_{TE}$ as a function of wavelength and spacer thickness ranging from 0.05 to 2.0 μm with a step of 10 nm, which is obtained by COMSOL Multiphysics [50]. A dip, due to the first order Wood–Rayleigh (WR) anomaly (or the first order surface plasmon resonance), is found in the colormaps of $T{\left(0\right)}_{TM}$ and PER(0) regardless of the spacer thickness (Note that T(±1)=0 when $\lambda >\lambda_{WR\left(1\right)}$, as shown in the lower panel of Figure A1).

It is commonly known that change in the grating period or grating width affects the polarization sensitivity performance of a single-layer grating (Figure A2, Figure A3, Figure A4 and Figure A5). As the grating period of the single-layer grating decreases, TM-transmission (*T*_S(0)_) is increased, while TE-transmission (*T*_S(90)_) is suppressed, thereby enhancing the polarization extinction ratio (PER_S_), as shown in Figure A2 (upper panel) and Figure A3 (solid line). It can also be observed in the upper panel of Figure A2 that a dip is shifted to a shorter wavelength as the grating period *p* decreases, which is well known as the feature of the first order Wood–Rayleigh (WR) anomaly, and thus the smaller grating period than ~*λ*_WR(1)_ / *n*_sub_ is needed to effectively design the single-layer grating structure *λ*_WR(1)_ is the wavelength of the first order WR and *n*_sub_ is the refractive index of the substrate). The complexity, difficulty, and cost are increased to fabricate the grating pattern with such small periodicity, however, the double-layer grating structure has the potential to reduce these drawbacks in the fabrication (lithography) by simply stacking two identical grating layers with a large grating period, as well as to improve the polarization sensitivity, as shown in the lower panel of Figure A2 and Figure A3 (dash-dot line) because a small amount of TE-transmission through the upper grating layer is further reduced by the lower grating layer and simultaneously TM-transmission is improved, due to the FP cavity resonance inside the spacer layer. For this reason, we used a relatively large grating period, *p* = 1 μm, to demonstrate high polarization sensitivity and incidence angle-insensitivity of a dielectric spacer bounded by nanoimprint-lithographically fabricated two gold grating layers. Similarly, the grating fill factor (i.e., the ratio of grating width to pitch) was fixed at 0.7, to have both high PER and practical linear polarizer (i.e., feasible, easy-to-fabricate), as shown in Figure A4 and Figure A5.

Aforementioned, a dip in the transmission spectra for the single- and double-layer grating structures with a pitch of 1 μm is observed in the wavelength range of interest (3–5 μm), which is attributed to the first order WR anomaly. This dip wavelength is found at $\lambda_{WR\left(1\right)}=n_{sub}\cdot p\approx$3.4 μm, where $n_{sub}$ is the refractive index of the substrate. Figure A6 shows the first diffracted transmission ($T_{tot}-T_{0}$) clearly below the wavelength of $\lambda_{WR\left(1\right)}$, and the transmitted light through the single- and double layer gratings is in the zeroth-order diffraction when $\lambda >\lambda_{WR\left(1\right)}$. Note that our double-layer grating was designed on the basis of Figure 1 and Figure A1 to have a peak wavelength of ~4.3 μm.

In order to the influence of alignment-shift of upper Au grating on the polarization sensitivity performance, we performed numerical simulations using COMSOL Multiphysics. We present the TM-, TE-transmission, and polarization extinction ratio using the colormap to show the spectral change clearly. The wavelength, and misalignment between upper and lower Au gratings, are varied continuously. The geometrical parameters of the double-layer grating are as follows: grating period (*p*) = 1 μm, grating width (*w*) = 0.7 μm, Au thickness (*t*_Au_) = 0.1 μm, spacer thickness (*t*_d_) = 0.25 μm. The lateral misalignment between two metal gratings is gradually varied from perfectly aligned (Case A: 0∙*p* = 0 nm) to completely alignment-shifted upper Au grating (Case D: *p*/2 = 500 nm), as displayed in Figure A7. For a minor misalignment, there is a slight difference in the simulation results (Figure A7 and Figure A8) as compared to the perfectly aligned double-layer grating. More interestingly, Figure A7 and Figure A8 show that the overall performance is improved as the lateral misalignment is increased, until the upper Au grating is perfectly misaligned (i.e., Case D). The configuration of Case D is similar to the bilayer grating structure.
